# The Role and Significance of Bioumoral Markers in Prostate Cancer

**DOI:** 10.3390/cancers13235932

**Published:** 2021-11-25

**Authors:** Traian Constantin, Diana Alexandra Savu, Ștefana Bucur, Gabriel Predoiu, Maria Magdalena Constantin, Viorel Jinga

**Affiliations:** 1 Faculty of General Medicine, “Carol Davila” University of Medicine and Pharmacy, 050474 Bucharest, Romania; traian.constantin@umfcd.ro (T.C.); gabriel.predoiu@drd.umfcd.ro (G.P.); viorel.jinga@umfcd.ro (V.J.); 2 Department of Urology, “Prof. Dr. Theodor Burghele” Hospital, 050659 Bucharest, Romania; 3 IInd Department of Dermatology, Colentina Clinical Hospital, 020125 Bucharest, Romania

**Keywords:** prostate cancer detection, screening, bioumoral markers

## Abstract

**Simple Summary:**

Prostate cancer (PCa) represents a very important health problem worldwide. Used as the main screening method for almost four decades, PSA (Prostate Specific Antigen) has proven its limitations. In this review, the authors try to make an evaluation of the biomarkers commercially available and used to improve the PCa detection in patients with elevated PSA. The authors also present the current PCa screening and diagnosis protocols in Romania.

**Abstract:**

The prostate is one of the most clinically accessible internal organs of the genitourinary tract in men. For decades, the only method of screening for prostate cancer (PCa) has been digital rectal examination of 1990s significantly increased the incidence and prevalence of PCa and consequently the morbidity and mortality associated with this disease. In addition, the different types of oncology treatment methods have been linked to specific complications and side effects, which would affect the patient’s quality of life. In the first two decades of the 21st century, over-detection and over-treatment of PCa patients has generated enormous costs for health systems, especially in Europe and the United States. The Prostate Specific Antigen (PSA) is still the most common and accessible screening blood test for PCa, but with low sensibility and specificity at lower values (<10 ng/mL). Therefore, in order to avoid unnecessary biopsies, several screening tests (blood, urine, or genetic) have been developed. This review analyzes the most used bioumoral markers for PCa screening and also those that could predict the evolution of metastases of patients diagnosed with PCa.

## 1. Introduction

Prostate cancer (PCa) was in 2020 the second most common cancer and the fifth leading cause of death among the male population worldwide, accounting for 6.8% of all male cancer mortality [1]. The highest incidence rates (between 83.4 and 62.5 per 100,000 men) were reported in Northern and Western Europe, the Caribbean, Australia/New Zealand, North America, Southern Africa, and South America, while the lowest rates were in Asia and North Africa [1]. In Eastern Europe, the incidence and mortality rates were 46.4/100,000 (ASR) and 13.7/100,000 (ASR) respectively [1]. In Romania in 2020, there was an incidence rate of PCa of 41.5/100,000 (ASR) with a mortality rate of 10.7/100,000 (ASR) [2]. The well-established risk factors are older age, family history of the disease, and black race or ethnicity [3]. In addition, genetic mutations, such as changes in the breast cancer gene 2 (BRCA2), checkpoint kinase 2 (CHEK2), ataxia telangiectasia mutated (ATM), and the breast cancer gene 1 (BRCA1) are present in diagnosed patients with PCa [4]. The presence of genetic conditions such as hereditary prostate cancer (HPC), hereditary breast and ovarian cancer syndrome (HBOC) or Lynch syndrome strongly recommends genetic testing in patients with suspicion of PCa [5]. Diet habits may play a role in PCa—a high consumption of tomatoes and lycopene, soy products, fish, green tea, and coffee having a prevention effect for PCa, while the abuse of grilled or fried meat, dietary supplements, and alcohol represent risk factors for PCa [6]. Patients with diabetes mellitus [7], especially those treated with metformin [8], have a lower risk of PCa, compared to the normal population. Exposure to Agent Orange (a herbicide extensively used in the Vietnam War), has been associated with a high risk of aggressive PCa [9]. PCa is linked with increased incidence and mortality in the heavy smoker population [10]. Inflammatory bowel disease [11] and gonorrhea infection [12] could also be considered risk factors for PCa.

The discovery of Prostate-Specific Antigen (PSA) in the late 1980s and early 1990s revolutionized the diagnosis and management of prostate cancer. Although PSA is not an ideal tumor marker, it is one of the most widely used markers in the diagnosis and follow-up of PCa [13]. PSA testing has limited specificity for the detection of PCa, which has led to unnecessary prostate biopsies (60–75%), especially in patients with total PSA levels between 4 and 10 ng/mL [14,15]. Elevated PSA levels are detected in 30–50% of cases of benign prostatic hyperplasia (BPH) and only 25% of cases are associated with PCa [16].

The correlation between PSA and PCa has decreased over the last 20 years [17]. Total PSA is now an important marker for prostate volume, growth, and results related to benign prostatic hyperplasia (BPH) [18,19]. PSA has a high negative predictive value if the value is below the maximum cut-off point of 4 ng/mL. There is an important unsatisfied need to find one or more biomarkers that will be able to correctly identify prostate cancer, and, more specifically, high risk prostate cancer [20].

An ideal biomarker should have a high sensitivity and specificity, it should be a non-invasive and inexpensive test that can accurately differentiate benign tissue from cancerous tissue and aggressive tumors [21]. In this review, we will highlight the novel biomarkers that show promise for early detection and management of prostate cancer (Table 1).

## 2. PSA and PSA Derivatives

### 2.1. Total PSA

PSA, also known as human kallikrein 3, is a blood-circulating glycoprotein which is bound to some protease inhibitors, including α1-antichymotrypsin and α2-macroglobulin. The FDA first approved Prostate-specific antigen screening in 1986 as a prognostic marker for PCa. The introduction of PSA in PCa screening and diagnosis represented a revolution in the disease management, with a significant increase of PCa’s incidence and prevalence due to patients’ diagnosis in earlier stages, resulting in a reduction in mortality rates [22].

Although PSA is not cancer-specific, it is organ-specific. Non-malignant diseases, such as prostatitis, BPH, urinary tract infections or iatrogenic maneuvers (prostate biopsy, bladder catheterization, and urethrocystoscopy) may increase PSA values [23].

**Figure 1:**
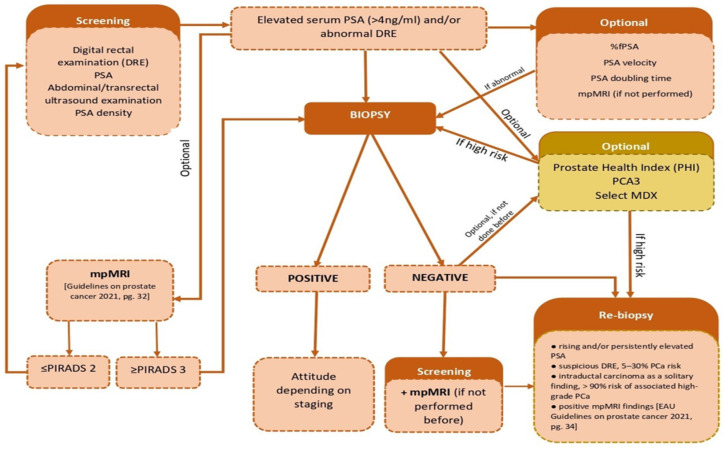
PCa screening and diagnosis protocol in Romania [26]. Abbreviations: PSA, Prostate Specific Antigen; %fPSA, free to total PSA ratio; PCA3, prostate cancer antigen 3; PHI, Prostate Health Index; mpMRI, multiparametric magnetic resonance imaging; PCa, prostate cancer; PIRADS, Prostate Imaging-Reporting and Data System.

The accepted normal range for tPSA is 0–4 ng/mL, although there is no general consensus on these values. In approximately 20–25% of cases with PCa, the tPSA value is less than 4 ng/mL; tPSA values of 4–10 ng/mL and over 10 ng/mL were observed in 67% of patients [24]. tPSA is important in evaluating the oncological treatment outcomes (surgery, radiotherapy, hormone therapy, chemotherapy) in patients confirmed with PCa [25]. In Romania, according to the Ministry of Heath guidelines, PSA detection is used for the PCa screening (Figure 1), but also for the surveillance of patients confirmed with PCa, regardless of the chosen treatment method [26].

Prostate biopsy is performed for clinical and/or biochemical suspicion of prostate cancer. Multiparametric magnetic resonance imaging (mpMRI) is usually recommended before biopsy, but is not a mandatory investigation. %fPSA, PSA velocity, and PSA doubling time are optional, but commonly used. In Romania, PHI, PCA3 and Select MDX tests are available in private clinics (performed before biopsy or as additional tests in patients with negative results, but with continuous PCa suspicion), but they are not used in common practice due to significant costs. If not performed before, mpMRI is strongly recommended for patients with previous negative biopsy and PCa suspicion.

### 2.2. Free PSA and Free/Total PSA Ratio

Serum PSA circulates in a higher percentage bound to one or several proteins, most commonly alpha-1-antichymotrypsin (ACT) and also in unbound “free” form [27]. Free PSA (fPSA) constitutes 5–40% of the total PSA. The f/tPSA ratio formula is expressed as %fPSA = 100 × fPSA/tPSA. Studies have shown that %fPSA is lower in men with prostate cancer than benign prostatic pathologies (especially BPH) [28]. Therefore %fPSA has shown the potential to discriminate between benign and malignant prostatic disease [28].

Especially in men with PSA levels in the “diagnostic grey zone” (PSA = 4.0 − 10.0 ng/mL), f/tPSA exceeded total PSA because it is more sensitive to PCa discrimination from benign tissues [29]. For a threshold value of 25%, f/tPSA, had a sensitivity of 95% and a specificity of 20% for PCa diagnosis in patients with a PSA value between 4 and 10 ng/mL [30]. The potential benefit of using %fPSA consisted in avoiding the unnecessary biopsies in patients undergoing prostate cancer evaluation [31].

One prospective multicenter study conducted in 1998 revealed that %fPSA decreased the rate of unnecessary biopsies by 20% when using a 25% cutoff point of the ratio. Another result of the study showed that cases with prostate cancer identified at a ratio of more than 25% were reduced in numbers, and, they had low risk prostate cancers [32].

According to the National Academy of Clinical Biochemistry (NACB), the use of %fPSA is recommended to differentiate patients with a suspicion of prostate cancer from those with BPH, when total serum PSA levels are between 4–10 ng/mL [33]. In our clinical practice this ratio has some utility to identify potential prostate cancer cases if the PSA is in the gray interval. This must be interpreted in a given clinical scenario and although it is not diagnostic, it offers some extra information to make a decision regarding the recommendation of prostate biopsy. The Romanian Ministry of Health includes %fPSA as an optional investigation for PCa screening [26].

### 2.3. Complexed PSA (cPSA)

Complexed PSA is found in serum in a conjugated form (60–90% bound to antichymotrypsin, 10–20% alpha-2-macroglobulin and 1–5% alpha-1-protease inhibitor) and constitutes 60–95% of total PSA [34]. Several studies have shown that cPSA enhances the specificity of tPSA in men with tPSA greater than 4 ng/mL and within clinically relevant tPSA ranges of 4–10 ng/mL and 2.5–4 mg/mL [35,36].

A prospective multicenter study, based on 831 patients showed that using a cutoff point of 2.5 ng/mL for total PSA and 2.2 ng/mL for Complexed PSA, with the cPSA range between 1.5 to 3.2 ng/mL provided a specificity of 21.2% and 35%, respectively, and a sensitivity of 85% for prostate cancer detection [36].

### 2.4. Age-Specific PSA

It has been shown in studies in the late 1990s that with age-related prostate growth (volume), there is a logarithmic increase in serum tPSA [37]. A further investigation of this finding showed that creating age-related cut-off points for total PSA may lead to an increased sensitivity of detecting prostate cancer in younger men, and reduce unnecessary prostate biopsy procedures in patients over 70 years of age [38]. Although there is some evidence to support the use of age-adjusted reference intervals, they are not frequently used in clinical practice and do not appear to have a standardized application. Further investigations are needed in this regard, but age-related PSA intervals appear to be an inexpensive way to add further value to PSA screening [39].

### 2.5. PSA Doubling Time (PSADT)

It is defined as the time required for serum PSA to double its value. The formula used to calculate PSADT IS [log(2)*T2 − T1(time difference)]/[log PSA2 − logPSA1]. It is considered to be more useful in the treatment stages and to monitor the recurrence of prostate cancer, rather than the diagnosis [40].

### 2.6. PSA Velocity (PSAV)

First described by Carter et al. in 1992, PSA velocity is defined as the absolute annual increase in PSA, measured in ng/mL/year [41]. At a cut-off point of ≥0.3–0.5 ng/mL/year the PSAV specificity was 90% in the monitoring of patients with prostate cancer, compared to 60% if total PSA was used [42]. Even successfully used in PCa prediction, the usefulness of PSAV in screening is still controversial [43]. PSAV has been shown to have little to no value as an independent predictor of a positive prostate biopsy result, as shown by The European Randomized Study of Screening for Prostate Cancer (ERSPC) [44].

### 2.7. PSA Density (PSAD)

PSAD, introduced in the early 1990s by Benson et al. has increased the accuracy of PSA diagnosis and is defined as the ratio of PSA concentration to prostate volume [45]. It is a better predictor of PCa than PSA, but has not been used in common practice over the years [46]. PSA density of the transition zone (PSADTZ), described by Kalish et al. had a better prediction for positive biopsy than conventional PSAD, in patients with PSA levels in the “grey zone” (4.0–10.0 ng/mL) [47]. Other studies have shown that PSADTZ was superior compared to PSAD in discriminating PCa from BPH [48]. Despite these positive findings, PSAD does not currently occur in clinical practice, although it is used in academic investigations.

### 2.8. ProPSA (pPSA)

ProPSA is a precursor of PSA that contains 244 amino acids released from peripheral malignant tissue rather than the hyperplastic areas developed in the transitional zone [49]. ProPSA has many subfractions, for example, [−2]Ppsa—described by Mikolajczyk et al., which was first found in prostate cancer tissue and has the most stable form in serum [50]. Pro-PSA is more often associated with peripheral zone cancer than transition zone hyperplasia [51].

A prospective study of 566 subjects diagnosed with PCa, detected on biopsy, showed that with a sensitivity of 80%, %[−2]proPSA had a higher specificity (51.6%), compared to PSA (29.9%) and %fPSA (28.9%) [52].

The use of tPSA and its derivatives for PCa detection is still common in most countries. Other blood and urine tests have been developed to avoid unnecessary prostate biopsies with a concurrent reduction in the risk of complications after this procedure (Table 2).

### 2.9. isoPSA

It is a well-established fact that PSA has multiple isoforms. Testing for PSA isoforms is a well-documented and possible choice when it comes to biomarkers that are involved in prostate cancer diagnosis and prognosis. Thus, testing for isoforms that we know of, for example PHI and 4KScore, is a common clinical tool to be used by the attending physician. The major problem with testing for only for what we know in terms of PSA isoforms is that they are only useful if they are present in that specific patient [53].

It is a well-established fact that proteins associated with cancer processes undergo significant structural changes due to point mutations, truncations, and post-translational modifications [54,55]. This will lead to altered metabolism of cancer cells and different protein interactions.

IsoPSA is a blood based, structure-oriented assay that predicts the risk of high-risk prostate cancer by partitioning the isoforms of prostate specific antigen that are associated with prostate cancer [56].

Clinical validation studies have shown that isoPSA structure assay is highly more effective than concentration-based PSA testing or free PSA to PSA ration in detecting high grade prostate cancer. isoPSA showed good values on ROC analysis with an AUC of 0.784 which was shown to be improved on subset analysis of MRI fusion guided prostate biopsy to an AUC of 0.831 [57].

## 3. Alkaline Phosphatase (ALP)

ALP is an enzyme that under physiological conditions can dephosphorylate compounds under alkaline conditions [58]. It is widely used in liver and bone diseases, and has been investigated as a potential biomarker in metastatic prostate cancer [59].

It has been postulated that ALP might be used as a biomarker in the setting of bone metastatic prostate cancer. A meta-analysis of prostate cancer studies which looked at the usefulness as a biomarker of alkaline phosphatase, included 63 studies (16,135 patients summed up) and showed that high levels of alkaline phosphatase were clearly associated with a poor overall survival and progression free survival, but found no statistical relationship with cancer specific survival [60].

In Romania, ALP is widely used for the surveillance of the treatment outcome in bone metastatic PCa.

## 4. Prostate Specific Membrane Antigen (PSMA)

PSMA is a transmembrane protein structure that is expressed in all forms of prostatic tissue, including carcinoma. It currently represents a very important target for the administration of personalized treatment of metastatic prostate cancer, by binding PSMA ligands to radionucleotides [61].

Retrospective analysis of prostate cancer samples for PSMA expression supports its utility as a prognostic tissue biomarker suggestive of lethal disease, correlating with higher Gleason score and PSA at diagnosis [62]. It has also been observed that PSMA expression is higher in lymph node metastasis of prostate cancer and has been associated with a reduced time to biochemical recurrence [63].

PSMA expression can be measured in circulating tumor cells and it has been associated with shorter progression, free survival, and overall survival and castrate-resistant metastatic disease [64]. Additionally, the overall expression of PSMA is considered to be a surrogate indicator of the poor response to the currently approved medication of metastatic Castration-Resistant Prostate Cancer (mCRPC) [64].

PSMA as a biomarker shows potential, especially in mCRPC settings. Additionally, it is a very important target and response to treatment in the upcoming field of theranostics [65].

## 5. Prostate Health Index (PHI)

Prostate Health Index (PHI) is a mathematical formula that combines three different forms of PSA: total PSA, free PSA, and [−2]proPSA [66]. PHI is calculated using the following equation: ([−2]proPSA/PSA free) × √PSA [67]. PHI was recognized by the FDA in June 2012 as an efficient clinical test for the early detection of prostate cancer in men >50 years of age, with PSA levels between 4.0 and 10.0 ng/mL and without any clinical modification on the digital rectal examination (DRE) of the prostate [68].

PHI shows to be superior to tPSA and %fPSA in predicting the presence of prostate cancer, including aggressive forms [69]. A meta-analysis of eight studies concluded that the PHI sensitivity for prostate cancer diagnosis was 90%, while the specificity was 31.6% [70]. In addition, PHI had a higher accuracy for prostate cancer detection, especially for patients with PSA levels between 2 ng/mL and 10 ng/mL [70].

PHI also proved to have a higher predictive accuracy for an aggressive disease (Gleason score ≤ 8) than PSA and % free PSA [71]. Loeb et al. reported that the PHI specificity was 36% compared to 17.2% for PSA and 19.4% for %fPSA in the detection of high-risk PCa [71]. For a cut-off value of 24, PHI measurement could spare 36–41% of unnecessary biopsies and 17–24% of over diagnosed low risk cancers [72].

The main clinical use of PHI is to avoid unnecessary biopsies in men with PSA levels in the “grey zone” and this value can range from 15% to 46%, depending on the cut-off point used [73]. Currently, PHI is not recommended to be used in the initial screening for PCa, but it may be considered in the next years as an efficient evaluation method for patients with PSA values between 2 ng/mL and 10 ng/mL [74].

Currently in Romania, PHI is not used in common practice and is available in selected private urology clinics.

## 6. Prostate Cancer Antigen 3 (PCA3)

Prostate cancer antigen 3 (PCA3), or DD3 (Differential Display Code 3), is a non-coding RNA produced almost exclusively in the prostate tissue. Bussemakers et al., who first identified and described it in 1999, also proved that the PCA3 gene is significantly overexpressed in malignant tissue compared to normal prostate [75].

In order to measure PCA3 mRNA, a urine sample obtained after a prostate massage is analyzed using real-time quantitative polymerase chain reaction (qRT-PCR) technique. This method is used because digital rectal examination induces pressure inside the prostate, resulting in the release of prostate cells through the prostate ducts and into the urethra [76]. The Progensa PCA3 test was approved by the FDA in 2012 and is a semi-automated assay that includes the isolation, amplification, hybridization, and quantification of PCA3 and PSA mRNAs using DTS systems [77].

A PCA3 score uses the ratio of PCA3 mRNA to PSA concentration (PCA3/PSA × 1000) [78]. The ideal cutoff score used for PCA3 is still controversial. The most appropriate cutoff for the PCA3 score recommended by the FDA is less than 25 and is associated with a low probability of a positive biopsy. Roobol et al. showed that PCA3 sensitivity significantly increased by lowering the cut-off values (68%, 84%, and 97% for the cut-offs of 35, 20, and 10, respectively) [79]. These authors also suggested that 26% of aggressive prostate cancer cases were missed using the cutoff of 35 [79]. Although its role in screening and surveillance remains unclear, PCA3 may serve as a valuable biomarker that goes further.

PCA3 mRNA detection is available in Romania.

## 7. Michigan Prostate Score (MiPS)

MiPS was developed by Tomlins et al. and the results were published in 2015 [80]. It is an assay that incorporates serum PSA level, urine PCA3 mRNA, and urine TMPRSS2-ERG [80]. The urine sample that is used in this test should be taken no later than 1 h after the digital rectal examination. The score is designed for men with high PSA levels with an indication of initial biopsy or for patients who are considering repeating the biopsy. A score between 1 and 100 reflects the percentage chance of prostate cancer on biopsy [81]. Therefore, while MiPS certainly provides value over PSA alone, its diagnostic value over PCA3 still requires validation [82].

The MiPS AUC was 0.751 compared to PSA, PSA plus T2:ERG score and PSA plus PCA3 score AUCs (0.585, 0.693 and 0.726 respectively, *p* value < 0.05) [80].

In an earlier study, Salami et al. showed that incorporating PSA, PCA3, and TMPRSS2-ERG in a multivariable algorithm is more specific than any of them as individual variables with a specificity of 90% and a sensitivity of 80% [77].

## 8. 4Kscore (Four Kalikrein Panel)

The 4Kscore represents a serum-based biomarker that associates multiple factors: age, digital rectal examination aspect, results of previous prostate biopsies, measurements of tPSA, free PSA, intact PSA, which is a form of free PSA, and human kallikrein 2 (hK2) that has promising results in PCa detection. [83]. A significant number of studies revealed that the 4Kscore is an important predictor of the diagnosis of intermediate and high-grade cancer (Gleason Score ≥ 7) in prostate biopsy [84]. At a PSA value higher than 3 ng/mL, the biomarker is more sensitive in the detection of high-grade prostate cancer than clinical variables individually [85]. A 4Kscore of ≥20 indicate high-risk disease and the necessity for prostate biopsy. On the other hand, values between 1–7.5 are considered low risk [86].

A prospective multi-institutional study conducted in the US, which included and evaluated 1012 patients undergoing prostate biopsy, confirmed the value of the 4Kscore [87]. At a cut-off value of 6%, approximately 30% of biopsies could be avoided, delaying the diagnosis for 1.3% of patients with high-grade prostate cancer [87].

A study based on 137 patients revealed that using 7.5% as a cutoff for the 4K score, the sensitivity of the multiparametric biomarker is 89% and the specificity is 29% [88].

Compared to PHI, a meta-analysis revealed that the accuracy of the diagnosis is similar for both tests [89]. It was established that the 4K score has a significant role in avoiding unnecessary prostate biopsies and has been reported to range from 49% to 57%, depending on the cut-off value used [90].

## 9. The Stockholm-3 Model, or STHLM3

The Stockholm-3 Model, or STHLM3, is a commercially available laboratory test, which is basically a statistical model that considers clinical, laboratory testing and genetic profile. Predictive factors used in the Stockholm-3 Model include clinical information (age, first degree—family history of prostate cancer and previous biopsy), blood biomarkers (total PSA, free PSA, free PSA to PSA ratio, hK2, macrophage inhibitory cytokine-1 (MIC 1) and β-microseminoprotein (MSMB)), genetic markers (a genetic score based on 254 single nucleotide polymorphism and a specific score for the HOXB13 SNP) and information from prostate examination (digital rectal examination and prostate volume measured using MRI examination) [91].

The main clinical utility of STHLM3 is to reduce the number of unnecessary prostate biopsies and increase diagnosis of clinically significant prostate cancer. The negative predictive value of STHLM3 was calculated at 92% in one study [92].

## 10. Androgen Receptor Variant 7 (AR V-7)

Androgen receptor variant 7 is the most identified mutation that will lead to castration resistant prostate cancer. Knowing the expression of Androgen Receptors variants is a useful clinical tool that can help guide the change of therapeutic agents during treatment sequencing.

AR-V7 can be identified by immunohistochemistry in prostate tissue or metastatic tumors. It was shown that androgen receptor splice variant 7 was commonly observed in metastatic tumors [93]. Given the practical and technical challenges associated to metastatic tumor biopsy, AR-V7 splice variants can be measured more easily by “liquid biopsy” [94]. In a peripheral blood sample, the expression of AR-V7 can be measured in circulating tumor cells.

Several studies have assessed the utility of AR-V7 as a biomarker for metastatic castrate resistant prostate cancer and it was considered useful for the decision making in regard to treatment sequencing [95].

## 11. Genetic Panels in Prostate Cancer Prognosis

Currently, genetic tests with an important role in the diagnosis and prognosis of the disease are used in common practice, but due to cost, complexity, and accessibility, they are not available for all categories of patients (Table 3).

The Prolaris test evaluates the expression of 31 genes that are part of a cell-cycle progression (CCP), an essential step in the cancer development and evolution and it represents a more potent prognostic factor than PSA [96]. The test generates a score with values from −3 to +3, based on modifications in gene expression levels. A higher score predicts adverse events in patients following treatment for PCa [97].

The Prolaris test, performed on tissue samples obtained after prostate biopsy or radical prostatectomy, could be useful in establishing the indication for active surveillance or active treatment in patients with low-risk PCa [98]. The test could also be useful for high-risk patients as it may suggest adjuvant therapy [98]. National Comprehensive Cancer Network (NCCN) guidelines recommend using the Prolaris test for very-low and low-risk PCa patients with positive biopsies and a life expectancy higher than ten years [99].

The Oncotype DX test (Genomic Prostate Score—GPS) is an analysis based on the detection of 17 gene panels using real-time PCR, performed on tissue samples obtained by prostate needle biopsy, prepared and fixed in paraffine [100]. GPS measures gene expression in prostate tumors using a scale of 0 to 100, a higher score indicating a more suggestive pathology. GPS can guide clinicians to separate patients for active surveillance from patients for therapeutic interventions [101]. The GPS score should be combined with other clinical parameters (age, PSA, clinical stage, and biopsy Gleason score) [102].

On radical prostatectomy, the Oncotype DX test predicted intermediate and high-grade (Gleason score ≥ 4 + 3) and high-stage (≥pT3) disease [103]. It has been shown to predict the aggressiveness of cancer on prostate needle biopsy [103]. More than 50% of the patients with unfavorable intermediate (UFI) risk PCa and GPS score above 40 will have biochemical recurrence at 10 years, while 25% will develop metastatic disease, compared to patients with scores below 40 [104]. The NCCN guidelines suggested that Oncotype DX could be used after biopsy, for patients with very-low and low-risk disease and high life expectancy (10 to 20 years) [105].

Decipher is a tissue-based genomic test that includes 22-gene-expression signatures identified and associated with aggressive PCa after prostate biopsy and/or radical prostatectomy. It was codeveloped and validated by GenomeDx Biosciences and Mayo Clinic and it is approved as a prediction tool for the risk of metastasis development after surgery. The test generates a risk score with values between 0 and 1 (with increments of 0.1) [106]. Decipher represents a significant prognostic factor for early metastatic disease and, for PCa specific death [106].

Dalela et al. developed a risk stratification tool (a score with values between 1 and 4) that helps to identify the patients who could benefit from adjuvant radiotherapy versus initial observation. It is based on the cumulative number of lymph node invasion, pT3b/T4 disease, pathological Gleason score 8–10 and Genomic Classifier Decipher >0.6 [107].

Adjuvant radiotherapy reduced the 10-year clinical recurrence rate only in patients with a risk score equal to or greater than 2, suggesting that patients with a higher decipher score and unfavorable pathological characteristics should be considered for adjuvant treatment [108]. NCCN guidelines recommend using Decipher in patients with positive surgical margins (R1), any pT3 disease, or rising PSA after radical prostatectomy [99].

A review of 42 studies concluded that the Decipher genomic classifier score higher than 0.6 after localized treatment is a strong predictive factor for biochemical failure (defined as two consecutive PSA values of more than 0.2 ng/mL in patients who underwent radical prostatectomy [109] or any increase in PSA greater than or equal to 2 ng/mL higher than the PSA nadir in patients with radiotherapy [110] and metastatic disease [111]. The NCCN guidelines for PCa (2022) recommend salvage radiotherapy with hormone therapy after radical prostatectomy for patients with a Decipher score higher than 0.6 [112].

Select MDx is a non-invasive post-DRE urine methylation test that is available in clinical practice and improves patient selection for initial biopsy. It measures urinary mRNA levels of the three-gene panel (HOXC6, DLX1, TDRD1), and higher levels are associated with an increased probability of aggressive PCa [113]. The test has 98% negative predictive value for a Gleason score ≥ 7 disease and 99% negative predictive value for a Gleason score ≥8 disease, while reducing unnecessary biopsies by up to 53% [114]. A low-risk SelectMDx score is correlated with a 90% probability of PCa and, respectively a 98% high-risk PCa absence [108].

In a more recent prospective study, based on 163 patients, Select MDx was associated with a sensitivity of 76.9%, a specificity of 49.6% and a negative predictive value of 82% in the detection of clinically significant prostate cancer. The study also suggested that a combination of the Select MDx test and MRI could improve the accuracy in the detection of prostate cancer [115].

In Romania, the Select MDX test is available, but the costs are not covered by the National Health Insurance System. Therefore, only limited number of patients can benefit from this analysis. In the Urology Department of “Prof. Dr. Theodor Burghele” Clinical Hospital (Bucharest, Romania), the Select MDX test was performed for 120 patients, aged between 45 and 60, with elevated PSA levels (maximum 10 ng/mL) and without any clinical modification on digital rectal examination of the prostate. The patients were informed about the possibility of having the test as alternative to prostate multiparameter MRI examination. For the patients from the SelectMDX test group, the preliminary collected data revealed that the unnecessary prostate biopsies were reduced by 25%. The data is still under analysis, further conclusions will be published in a dedicated study.

## 12. Conclusions

**Figure 2:**
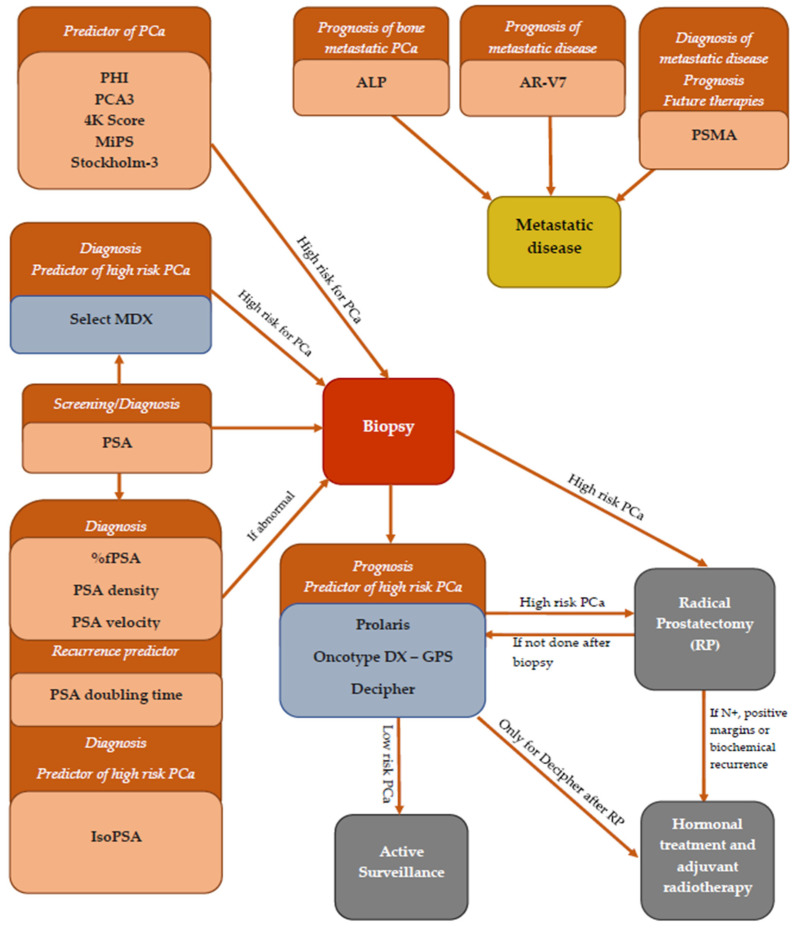
Summary of the most common PCa biomarkers and their use.

In this review, the authors have highlighted some of the numerous promising biomarkers commercially available and used to improve the PCa detection in patients with elevated PSA. Although PSA is currently the most broadly prostate cancer biomarker used for screening, other tests have been introduced into common practice and are being performed more and more extensively (Figure 2).

Complex tests such as PHI, 4Kscore, Stockholm-3 (based on PSA isoforms), PCA3, MiPS (based on PCA3) and Select MDX (genetic test) have an important role in stratifying patients with high risk for PCa, reducing at the same time the number of unnecessary biopsies.

Prolaris, Oncotype DX—GPS and Decipher are essential in the classification of patients diagnosed with PCa in the group of low-risk disease, with the recommendation for active surveillance or in the one with aggressive tumors, which will rapidly need active treatment, even (for Decipher test) adjuvant therapy after surgery.

PSMA and AR-V7 have significant role in the prediction and diagnosis of metastatic PCa, while “old, but very valuable” ALP is an essential marker for bone metastasis.

The management of patients diagnosed with PCa is well defined. However, overdiagnosis and overtreatment of PCa, early detection of the patients with a high-risk disease and early treatment in that category are still important challenges for health systems in all countries, and the extensive use of biomarkers in the diagnosis and prognosis of PCa could be an answer in solving these issues.

In recent years there have been great discoveries in the research of prostate cancer biomarkers; however, further studies are needed to define the appropriate context for their use. Ongoing investigation of these markers and new markers in the future will improve the detection and management of early prostate cancer, providing more accurate results.

## Figures and Tables

**Table 1:** Prostate cancer biomarkers used in clinical practice.

Biomarker	Role	Sample
Prostate Specific Antigen (PSA)	Screening Diagnosis	Blood
Free/Total PSA ratio (%fPSA)	Diagnosis	Blood
PSA density	Diagnosis	Blood
PSA velocity	Prognosis	Blood
PSA doubling time	Recurrence predictor	Blood
Iso PSA	Diagnosis Predictor of high risk PCa	Blood
Alkaline Phosphatase (ALP)	Prognosis of bone metastatic PCa	Blood
Prostate specific membrane antigen (PSMA)	Diagnosis of metastatic disease Prognosis Future therapies	Tissue Blood (circulating tumor cells)
Prostate Cancer Antigen 3 (PCA3)	Diagnosis	Urine
Androgen receptor variant 7 (AR V-7)	Prognosis of metastatic disease	Tissue Blood (circulating tumor cells)

**Table 2:** Serum and urinary biomarkers that may increase the accuracy of prostate cancer diagnosis.

Test	Analytes Detected	Fluid
Prostate Health Index (PHI)	PSA, fPSA, [−2]ProPSA	Serum
Prostate cancer antigen (PCA3)	PCA3	Urine collected after prostate massage
Four-kallikrein panel (4K Score)	PSA, fPSA, iPSA, khK2	Serum or plasma
MiPS	PCA3, TMPRSS2-ERG	Urine
Stockholm-3 (STHLM3)	PSA, fPSA, hK2, MIC 1, MSMB, genetic markers	Serum

Abbreviations: fPSA, free PSA; [−2]ProPSA, ProPSA; hK2, human kallikrein 2; iPSA, intact PSA; PCa, prostate cancer; PCA3, prostate cancer antigen 3; PHI, Prostate Health Index; PSA, Prostate Specific Antigen; MiPS, Mi-Prostate Score; TMPRSS2-ERG—The transmembrane protease serine 2:v-ets erythroblastosis virus E26 oncogene homolog gene fusion; MIC 1, macrophage inhibitory cytokine-1; MSMB, β-microseminoprotein.

**Table 3:** Genetic tests used in the prognosis of PCa.

Test	Role	Sample
Prolaris	Prognosis Predictor of high risk disease	Tissue
Oncotype DX—GPS	Prognosis Predictor of high risk disease	Tissue
Decipher	Prognosis Predictor of high risk disease Indicator for treatment	Tissue
Select MDX	Diagnosis Predictor of high risk disease	Urine
